# Prognostic Significance of the PROFUND Index on One Year Mortality in Acute Heart Failure: Results from the RICA Registry

**DOI:** 10.3390/jcm11071876

**Published:** 2022-03-28

**Authors:** Manuel Méndez-Bailon, Rosario Iguarán-Bermudez, Francesc Formiga-Pérez, José Carlos Arévalo Lorido, Iván Suárez-Pedreira, Jose Luis Morales-Rull, Ana Serrado-Iglesias, Pau Llacer-Iborra, Gabriela Ormaechea-Gorricho, Francisco Javier Carrasco-Sánchez, Jesús Casado-Cerrada, Emmanuel Andrès, Jesús Diez-Manglano, Noel Lorenzo-Villalba, Manuel Montero-Pérez-Barquero

**Affiliations:** 1 Internal Medicine Department, Hospital Clínico San Carlos, Universidad Complutense de Madrid, Instituto de Investigación Sanitaria del Hospital Clínico San Carlos (IdISSC), 28040 Madrid, Spain; manuelmenba@hotmail.com (M.M.-B.); draiguaran@gmail.com (R.I.-B.); 2 Internal Medicine Department, Hospital Universitari de Bellvitge, 08907 Barcelona, Spain; fformiga@bellvitgehospital.cat; 3 Internal Medicine Department, Hospital Universitario de Badajoz, 06080 Badajoz, Spain; joscarlor@gmail.com; 4 Internal Medicine Department, Hospital Valle del Nalón, 33920 Langreo, Spain; ivasuaped@yahoo.es; 5 Internal Medicine Department, Hospital Universitario Arnau de Villanova, 25198 Lleida, Spain; jl.morales@ono.com; 6 Internal Medicine Department, Hospital de Badalona, 08911 Badalona, Spain; aserrado@bsa.cat; 7 Internal Medicine Department, Hospital Universitario Ramón y Cajal, IRYCIS, 28034 Madrid, Spain; paullacer@hotmail.com; 8 Unidad Multidisciplinar de Insuficiencia Cardíaca, Hospital de Clínicas Dr. Manuel Quintela, Montevideo 11600, Uruguay; gabiorma@gmail.com; 9 Internal Medicine Department, Hospital Juan Ramón Jiménez de Huelva, 21005 Huelva, Spain; franciscoj.carrasco.sspa@juntadeandalucia.es; 10 Internal Medicine Department, Hospital Universitario de Getafe, 28905 Madrid, Spain; casadocerrada@telefonica.net; 11 Service de Médecine Interne, Diabète et Maladies Métaboliques, Hôpitaux Universitaires de Strasbourg, 67000 Strasbourg, France; emmanuel.andres@chru-strasbourg.fr; 12 Internal Medicine Department, Hospital Universitario Royo Villanova, 50015 Zaragoza, Spain; jdiez@aragon.es; 13 Internal Medicine Department, IMIBIC/Hospital Universitario Reina Sofía, Universidad de Córdoba, 14004 Córdoba, Spain; montero.manolo@gmail.com

**Keywords:** heart failure, comorbidities, PROFUND index

## Abstract

Background: Heart failure (HF) is a syndrome with high prevalence, mainly affecting elderly patients, where the presence of associated comorbidities is of great importance. Methods: An observational study from a prospective registry was conducted. Patients identified from the National Registry of Heart Failure (RICA), which belongs to the Working Group on Heart Failure and Atrial Fibrillation of the Spanish Society of Internal Medicine (SEMI), were included. The latter is a prospective, multicenter registry that has been active since 2008. It includes individual consecutive patients over 50 years of age with a diagnosis of HF at hospital discharge (acute decompensated or new-onset HF). Results: In total, 5424 patients were identified from the registry. Forty-seven percent were men and mean left ventricular ejection fraction (LVEF) was 51.4%; 1132 had a score of 0 to 2 according to the PROFUND index, 3087 had a score of 3 to 6, and 952 patients had a score of 7 to 10 points. In the sample, 252 patients had a score above 11 points. At the end of the year of follow-up, 61% of the patients died. This mortality increased proportionally as the PROFUND index increased, specifically 75% for patients with PROFUND greater than 11. The Kaplan-Meier survival curve shows that survival at one year progressively decreases as the PROFUND index value increases. Thus, subjects with scores greater than seven (intermediate-high and high-risk) presented the worst survival with a log rank of 0.96 and a *p* < 0.05. In the regression analysis, we found a higher risk of death from any cause at one year in the group with the highest risk according to the PROFUND index (score greater than 11 points (HR 1.838 (1.410–2.396)). Conclusions: The PROFUND index is a good index for predicting mortality in patients admitted for acute HF, especially in those subjects at intermediate to high risk with scores above seven. Future studies should seek to determine whether the PROFUND index score is simply a prognostic marker or whether it can also be used to make therapeutic decisions for those subjects with very high short-term mortality.

## 1. Introduction

Heart failure (HF) is a syndrome with high prevalence, mainly affecting elderly patients, where the presence of associated comorbidities is of great importance [1]. These comorbidities can make treatment more difficult and will have an important impact on prognosis, leading to more admissions, poorer quality of life, and contributing to increased mortality. HF patients usually present several associated comorbidities, while having no comorbidities in HF is the exception [2].

Most HF registries tend to include comorbidities that have a pathogenic relationship with HF and cardiovascular risk, such as arterial hypertension, diabetes mellitus, hypercholesterolemia, renal failure, smoking, chronic obstructive pulmonary disease, or atrial fibrillation. Other conditions or comorbid processes that appear to be unrelated to the HF process, such as cognitive deterioration, functionality, degenerative osteoarticular pathology, neoplastic processes, fragility, or socio-familial variables, can also directly influence the prognosis of these patients [3].

The RICA registry analyzed the prognostic value of baseline functional status, assessed by the Barthel index, showing a high prevalence of functional deterioration, with severe dependence for basic activities of daily living in 55.9% of patients. The Barthel index prior to admission was shown to be a prognostic predictor of prognosis [4]. The Barthel index is an ordinal scale used to measure performance in activities of daily living (ADL). Each performance item is rated on this scale, with a given number of points assigned to each level or ranking. It uses ten variables describing ADL and mobility. A higher number is associated with a greater likelihood of being able to live at home with a degree of independence following discharge from hospital.

Heart failure is one of the leading causes of death in the general population, but the prognosis varies widely depending on the patient. Quantification of the risk of these patients could improve and individualize the treatment, as well as establish a management care plan [5].

In recent years, the PROFUND index, a specific tool for estimating the mortality risk at one year in pluripathology patients, has been developed and was validated in a hospital-based, multicenter cohort, recruited in 36 Spanish hospitals. The index stratifies patients with multiple pathologies into four risk groups according to the score ranges obtained, with mortality ranging from 12 to 14% in the lowest risk stratum to 61 to 68% in those with 11 or more points. A total of 1632 patients were included and followed for one year and the index was subsequently derived and validated [6]. However, it is not known whether this index can be useful in patients with acute HF admitted to our departments. The aim of this study was to determine the clinical characteristics of patients with HF included in the RICA registry and to stratify them according to the PROFUND index into low, intermediate, or high risk, as well as to evaluate how the index predicts mortality at 1 year.

## 2. Methods

An observational study from a prospective registry was conducted. The patients from the National Registry of Heart Failure (RICA) belonging to the Working Group on Heart Failure and Atrial Fibrillation of the Spanish Society of Internal Medicine (SEMI) were included. The latter is a prospective, multicenter registry that has been active since 2008. It includes consecutive individual patients over 50 years of age with a diagnosis of HF at hospital discharge (acute decompensated or new-onset HF), according to European cardiology guidelines. Fifty centers included at least 20 patients. Subjects are included in the registry after hospital discharge and followed for at least one year. A total of 1657 patients were excluded from the analysis because they had not completed one year of follow-up, as well as patients with missing variables to calculate the PROFUND index and those dying during hospitalization, as the main objective of our study was to determine the prognostic impact of the PROFUND index and mortality at one year of follow-up.

The registry protocol was initially approved by the Ethics Committee of the Hospital Universitario Reina Sofía de Córdoba and was subsequently approved by each of the committees of the participating hospitals (code 18/349-E, last updated on 9 August 2018). All patients signed an informed consent form prior to inclusion in the registry. The data were collected from a web page (www.registrorica.org, accessed on 1 January 2008) containing the anonymous database and accessed by each investigator through a personalized password.

In the present analysis, we included patients older than 65 years registered from January 2008 to December 2020. We used personal history, physical examination, and clinical analysis records. Renal evaluation (glomerular filtration rate) was assessed through the CKD-EPI formula with chronic renal failure defined as CKD-EPI < 60 mL/min. Left ventricular ejection fraction (LVEF) was assessed by 2D echocardiography. Charlson comorbidity index and Pfeiffer test were also collected. The Charlson comorbidity index predicts the one-year mortality for a patient who may have a range of comorbid conditions, such as heart disease, AIDS, or cancer (a total of 22 conditions). Each condition is assigned a score of 1, 2, 3, or 6, depending on the risk of dying associated with each one. Scores are summed to provide a total score to predict mortality. The Pfeiffer test is a short, reliable instrument to detect the presence of intellectual impairment and to determine the degree, if any. Patients who did not have a complete blood count at discharge and 3 months after the acute HF episode, those who did not complete follow-up at 1 year, and those who died during hospitalization were excluded from the analysis.

Patients were classified according to the PROFUND index into four groups. This index stratifies 12-month mortality and it is based on nine clinical, analytical, and socio-familial dimensions. The index stratifies chronic patients with two or more chronic diseases into four risk groups, according to the score ranges obtained (Table 1) [7].

Legend: NYHA, New York Heart Association Classification; mMRC: modified Medical Research Council (mMRC) dyspnea scale; Hb: Hemoglobin. Patients admitted for acute HF were classified as follows.

1. Low-risk PROFUND group: with values between 0 and 2
2. PROFUND group low intermediate risk: with values between 3 and 6
3. PROFUND group high intermediate risk 7–10
4. High risk PROFUND group with scores greater than or equal to 11

Quantitative variables are expressed as mean (standard deviation) and qualitative variables as absolute values (percentages). Quantitative variables were compared using ANOVA and qualitative variables were compared using the Chi-square test. A post-hoc Tukey method was used. Kaplan-Meier curves were constructed, comparing the groups using the log-rank test. A bivariable analysis was performed between the clinical, analytical, therapeutic, and prognostic characteristics between the different groups. The primary endpoint of the study was all-cause mortality at 1 year. Secondary variables included mortality at 6 months and readmission rate for HF at 30 days, 3 months, and 1 year of follow-up. Subsequently, a Cox regression analysis was performed using the conditional forward method and 95% confidence intervals. In all cases, the level of statistical significance was set at *p* < 0.05. Statistical analysis was performed using the Statistical Package for Social Sciences (version 21.0, SPSS Inc., Chicago, IL, USA).

## 3. Results

During the study period, 5424 patients were discharged from the registry. Forty-seven percent were men and mean left ventricular ejection fraction (LVEF) was 51.4%. Table 2 shows the most notable demographic characteristics of the cohort, grouped according to their evolution with respect to the categories defined based on the PROFUND index.

Of the patients included, 1132 had a score of 0 to 2 according to the PROFUND index, 3087 had a score of 3 to 6, and 952 patients had a score of 7 to 10 points. In the sample, 252 patients had a score above 11 points. This last group of patients with HF had a higher mean age, was predominately women, and showed greater associated comorbid conditions, especially arterial hypertension, chronic renal failure, anemia, and cognitive impairment. Consequently, the Charlson index in this group of patients, stratified as high risk according to the PROFUND index, presented a greater degree of disability as evaluated by the Barthel index, higher scores in the Pfeiffer scale, and higher Charlson index relative to the lower risk groups (Table 2). Differences in NT-proBNP values did not reach statistical significance, although there was a tendency to present higher values in those patients with a higher PROFUND index. The evolution of HF in the higher risk group also appeared to be more prolonged, with 73% of patients in this group having been previously admitted.

At the end of the 1 year of follow-up, 61% of the patients had died. This mortality increased proportionally as the PROFUND index increased, specifically being 75% for patients with PROFUND greater than 11. The same trends, with statistically significant differences, were observed for death and readmission at 30 days and 6 months in favor of the group with the highest PROFUND index score. (Table 2).

**Figure 1:**
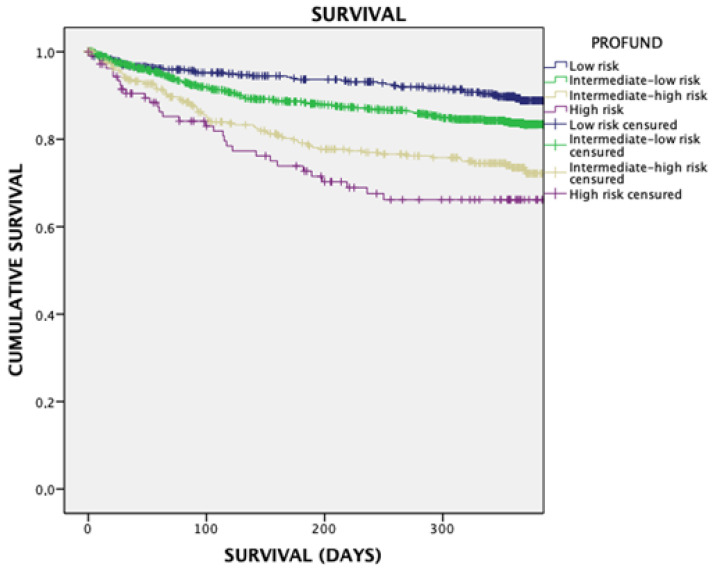
Kaplan-Meier curve for annual mortality in patients who have been classified by the PROFUND index.

The Kaplan-Meier survival curve shows that survival at one year progressively decreases as the PROFUND index value increases. Thus, subjects with scores greater than seven (intermediate–high and high-risk) presented the worst survival, with a log rank of 0.96 and a *p* < 0.05. (Figure 1)

In the regression analysis, we found a higher risk of death from any cause at one year in the group with the highest risk according to the PROFUND index (score greater than 11 points (HR 1.838 (1.410–2.396)). This HR obtained was higher than that of the Charlson index and the NT-proBNP and left ventricular ejection fraction values. The variables evaluated in the regression analysis are shown in Table 3.

Indeed, patients who died had a significantly higher ProBNP (8084/9863 vs. 6131/13,400, *p* < 0.01). The mortality ratio was available in 1419 cases, 82.2% of deaths were due to cardiovascular causes (Supplementary Table S1). The PROFUND index was also associated with cardiovascular mortality (intermediate high risk 254 (26.3), high risk 83 (32.8), intermediate low risk 520 (16.4), low risk 117 (9.4), *p* < 0.001). We also compared the PROFUND index only with the MAGGIC index, which is validated for one-year follow-up. The area under the curve is very similar; PROFUND Index: 0.624 and MAGGIC: 0.645. (Supplementary Figures S1 and S2).

## 4. Discussion

The evaluation of this cohort of more than 5000 patients demonstrates the importance of the PROFUND index in the prediction of mortality at one year in patients with heart failure admitted to internal medicine departments. These results are in line with those observed in comorbid patients admitted to internal medicine services, where the PROFUND index was also a good predictor of mortality at one year. These same findings have also been observed in cardiology and primary care settings [8,9].

Although there are already highly validated HF prediction models such as the MAGGIC scale or the MEESI scale for HF patients [5,10], we believe that the PROFUND index could be an easy-to-use tool to be implemented in clinical practice. Advanced age over 85 years, the presence of renal failure, anemia, and functional disability assessed by Barthel were variables included in the index that have been more prevalent in patients with HF and are associated with higher mortality in our cohort [4]. All of these factors are well known to be associated with a worse prognosis in HF [11,12]. We have observed a correlation between NYHA III-IV and higher PROFUND index scores, which result in worse prognosis. In addition, the burden of comorbid conditions also increased as the score increased. Chronic renal failure was more prevalent in patients with high PROFUND scores.

In our study, we did not obtain any statistically significant differences for NT-proBNP values, although there is a tendency to present higher values for subjects with higher mortality and PROFUND index. These findings have been observed in other studies evaluating both prognostic factors for 30 day mortality [13], and are also reflected in the adjustment of the multivariate model in our results. Left ventricular ejection fraction was not a predictor of 1 year mortality in our study, while the PROFUND index was a predictor, especially for those individuals with scores above seven. The study demonstrated the prognostic importance of extracardiac factors in the survival of patients with advanced HF [3].

The PROFUND index has been found to be higher in those patients with acute heart failure, with higher mortality rates at 1 year but also short-term mortality in the first 30 days and at 6 months of follow-up. These findings have been found for 1 year mortality in the Kaplan-Meier analysis. The PROFUND index is a useful tool not only for chronic patients with multimorbidity, but also for patients with acute heart failure admitted to internal medicine services in our country [14,15].

The study has some limitations: the evaluation of the PROFUND index has been performed on a retrospective cohort of patients with acute HF, so it would be useful to evaluate this index in prospective registries. In this sense, the Spanish Society of Internal Medicine is carrying out a prospective registry for patients with acute HF called PROFUND-IC with favorable preliminary results. The social and economic background of the patient has not been evaluated, so we lack data that may have an unfavorable impact on the evolution of the patient with HF.

## 5. Conclusions

The PROFUND index is a good index for predicting mortality in patients admitted for acute HF, especially in those subjects at intermediate to high risk with scores above seven. It would also be important to establish in future studies whether the PROFUND index score is simply a prognostic marker or whether it can also be used to make therapeutic decisions for those subjects with very high short-term mortality.

## Figures and Tables

**Table 1:** PROFUND index. Clinical variables included and scoring of these variables.

PROFUND Index	
VARIABLE	POINTS
age ≥ 85 years	3
Clinical features	
Active neoplasia	6
Dementia	3
III-IV NYHA dyspnea or 3–4 mMRC	3
Delirium during last hospital admission	3
Hb < 10 g/dL	3
Socio-familial situation	
Barthel index < 60	4
Absence of caregiver or other than spouse	2
≥4 hospital admissions over the last 12 months	3

**Table 2:** Baseline characteristics of patients with heart failure according to the PRFUND index.

Variable	All (N = 5424)	PROFUND 0–2 (N = 1132)	PROFUND 3–6 (N = 3087)	PROFUND 7–10 (N = 952)	PROFUND = o > 11 (N = 253)	*p* Value
Age media (sd)	79.9 (8.7)	71.2 (7.3)	81.4 (7.8)	83.9 (6.2)	85.0 (4.9)	<0.001
Sex. men, N (%)	2670 (47.3)	729 (58.3)	1506 (47.4)	346 (35.8)	89 (35.2)	<0.001
**Comorbidities**						
Hypertension N (%)	4866 (86.2)	1022 (81.2)	2760 (86.9)	856 (88.6)	228 (90.1)	<0.001
T2DM N (%)	2614 (46.3)	604 (48.3)	1404 (45.5)	436 (45.9)	122 (48.6)	0.317
COPD N (%)	1269 (23.4)	264 (23.4)	719 (23.3)	221 (23.3)	62 (24.5)	0.980
OSA N (%)	2223 (41.1)	331 (29.3)	1342 (43.5)	424 (44.6)	142 (56.1)	<0.001
Renal insufficiency N (%)	292 (5.4)	38 (4.3)	77 (2.5)	77 (2.5)	100 (39.5)	<0.001
Dementia N (%)	960 (17.7)	68 (6)	527 (17.1)	237 (24.9)	113 (44.8)	<0.001
Barthel (N = 4664) media (SD)	82.9 (22.4)	96.3 (7.8)	89.7 (13.0)	55.0 (23.8)	51.5 (26.1)	<0.001
Pfeiffer(N = 4183) media (SD)	1.60 (2.12)	0.62 (1.2)	1.31 (1.7)	3.02 (2.5)	4.31 (3.1)	<0.001
Familial support N (%)	5114 (94.3)	1048 (92.6)	2941 (95.3)	892 (93.7)	230 (91.3)	<0.001
Charlson score media (SD)	3.04 (2.5)	2.49 (2.2)	2.90 (2.4)	3.64 (2.7)	5.30 (3.2)	0.001
**NYHA N (%)**						
**I**	417 (8)	187 (16)	193 (6.2)	29 (3)	3 (1)	<0.001
**II**	2969 (55)	945 (83)	193 (6.2)	343 (36)	46 (18)	<0.001
**III**	1876 (35)	-	1194 (39)	523 (55)	188 (74)	<0.001
**IV**	162 (3)	-	95 (3)	57 (6)	16 (6)	<0.001
LVEF media (SD)	51.4 (15.7)	49.1 (16.4)	51.3 (15.3)	55.9 (15.7)	53.0 (15.1)	<0.001
BMI media (SD)	29.2 (7.5)	30.2 (9.4)	28.9 (7.3)	28.9 (5.6)	28.4 (6.0)	<0.001
Atrial fibrillation N (%)	2886 (53.2)	534 (47.2)	1664 (53.9)	553 (58.1)	143 (56.5)	<0.001
Ischemic cardiopathy N (%)	1393 (25.7)	303 (26.8)	793 (25.7)	239 (25.1)	60 (23.7)	0.677
Valvulopathy N (%)	889 (6.4)	137 (12.1)	537 (17.4)	178 (18.7)	43 (17)	<0.001
Previous HF	3335 (61.5)	578 (51.4)	1913 (62)	994 (69.8)	185 (73.1)	<0.001
**Laboratory N (%)**						
Hemoglobin (g/dl) media (SD)	12.0 (2.0)	13.0 (1.7)	12.0 (2.0)	11.5 (1.9)	10.5 (2.0)	<0.001
Glomerular filtration rate (ml/min) media (SD)	58.6 (26.4)	67.3 (27.8)	53.5 (25.4)	53.6 (25.4)	51.3 (26.5)	<0.001
proBNP (N = 2569) pg/mL media	6686.6	5829.4	6996.6	7500.0	8219.0	0.131
Albumin (N = 183) (g/L) media/sd	3.3 (0.6)	3.6 (0.7)	3.3 (0.6)	3.2 (0.6)	3.1 (0.6)	0.136
**Treatment N (%)**						
Beta blockers N (%)	36,883 (68.5%)	837 (74.4%)	2130 (69.4%)	561 (59.5%)	156 (62.8)%	<0.001
ACE inhibitors N (%)	2061 (38.3%)	486 (43.1%)	1111 (36.7%)	352 (37.1%)	98 (39.1%)	0.001
ARA-2 N (%)	1464 (27.4%)	305 (27.3%)	833 (27.1%)	266 (28.8%)	65 (26.1%)	0.720
Sacubitril valsartan N (%)	141 (2.6%)	38 (3.4%)	86 (2.8%)	15 (1.6%)	30 (1.2%)	0.025
Furosemide (mg) N (%)	64.3 (41.3)	61.8 (37.2)	64.8 (44.5)	64.6 (35.4)	68.7 (39.9)	0.087
Mineralocorticoids N (%)	1247 (23.1%)	271 (24%)	710 (23.4%)	199 (21.4%)	53 (21.3%)	0.447
SGLT2I N (%)	32 (0.6%)	12 (1.1%)	12 (0.4%)	4 (0.5%)	303 (1.2%)	0.020
Ivabradine N (%)	65 (1.2%)	19 (1.7%)	37 (1.2%)	7 (0.8%)	101 (0.4%)	0.171
Anticoagulant N (%)	2223 (40.6%)	441 (39.6%)	1265 (41.5%)	380 (40.2)%	88 (35.2%)	0.189
**Mortality at 30 days** N (%)	1611 (29.7)	217 (19.2)	866 (28.1)	405 (42.5)	123 (48.6)	<0.001
**30 days readmission** **N (%)**	1226 (22.8)	193 (17.1)	646 (21.1)	290 (30.7)	97 (38.6)	<0.001
**Mortality at 6 months** **N (%)**	1827 (33.7)	271 (23.9)	977 (31.6)	446 (46.8)	133 (52.6)	<0.001
**6 months readmission** **N (%)**	2120 (39.4)	363 (32.2)	1154 (37.7)	469 (49.6)	134 (53.4)	<0.001
**One year mortality** **N (%)**	3308 61.3%	645 57.1%	1821 (59.4)	647 (68.9)	189 (74.7)	<0.001
**One year mortality** **N (%)**	1478 (72.5)	781 (69.4)	2193 (71.6)	722 (76.4)	209 (83.3)	<0.001

Legend: T2DM: type 2 Diabetes Mellitus; COPD: Chronic obstructive pulmonary disease; OSA: obstructive sleep apnea; NYHA: New York Heart Association functional class; BMI: Body mass index; ACE inhibitors: angiotensin converting enzyme inhibitors; ARA-2: Angiotensin II receptor antagonists; SGLT2: Sodium Glucose Co-transporter Type 2.

**Table 3:** COX regression analysis of mortality at one year.

	Univariable		Multivariable	
Variable	RR (IC al 95%)	*p*	RR (IC al 95%)	*p*
PROFUND index Intermediate-Low Intermediate-High High Sex (Men)	1.648 (1.074–2.530) 2.619 (1.583–4.334) 2.525 (1.255–5.081) 1.086 (0.812–1.453)	0.022 <0.001 <0.001 0.577	1.703 (1.115–2.600) 2.712 (1.658–4.435) 2.590 (1.293–5.190)	0.014 <0.001 <0.01
Charlson score	1.082 (1.026–1.140)	0.004	1.089 (1.036–1.145)	0.001
proBNP/1000 pg/mL Gromerular filtration rate	1.018 (1.000–1.037) 0.997 (0.991–1003)	0.052 0.370	1.019 (1.002–1.037)	0.03
Left ventricular ejection fraction	1.003 (0.993–1.013)	0.513		

## Data Availability

Data Availability Statements in section “MDPI Research”.

## Supplementary Materials

The following supporting information can be downloaded at: https://www.mdpi.com/article/10.3390/jcm11071876/s1. Table S1: Causes of death; Figure S1: ROC curve of PROFUND Index; Figure S2: ROC MAGGIC CURVE.
