# Supplementary material for: Prognostic Significance of the PROFUND Index on One Year Mortality in Acute Heart Failure: Results from the RICA Registry
Source: J Clin Med. 2022 Mar 28;11(7):1876. doi: 10.3390/jcm11071876 (PMC9000036; doi:10.3390/jcm11071876)
Supplement: Supplementary file 1 [file jcm-11-01876-s001.zip › jcm-1602222-supplementary.pdf]

Supplementary Table S1. Causes of death

| Cause of death | Frequency | Percentage |
|---|---|---|
| Heart failure | 510 | 35.9 |
| Other cause | 202 | 1.2 |
| Refractory heart failure | 190 | 13.4 |
| Infection or sepsis | 182 | 12.8 |
| Sudden cardiac death (unexpected death in a natural way with no apparent cause). | 100 | 7.0 |
| Neoplasia | 61 | 4.3 |
| Ictus | 60 | 4.2 |
| Other vascular causes | 51 | 3.6 |
| Acute myocardial infarction | 36 | 2.5 |
| Death within 1 hour after symptom onset | 15 | 1.1 |
| Pulmonary embolism | 7 | 0.5 |
| After cardiovascular surgery | 3 | 0.2 |
| Aortic aneurism | 2 | 0.1 |

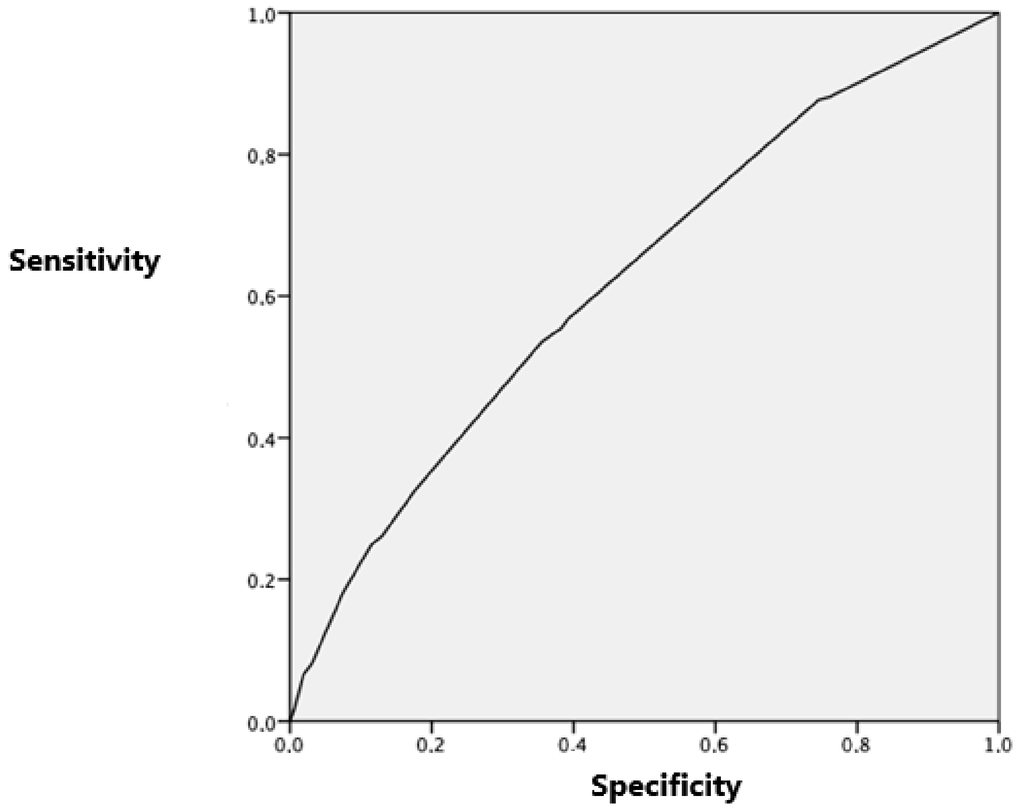


Figure S1. ROC curve of PROFUND Index.

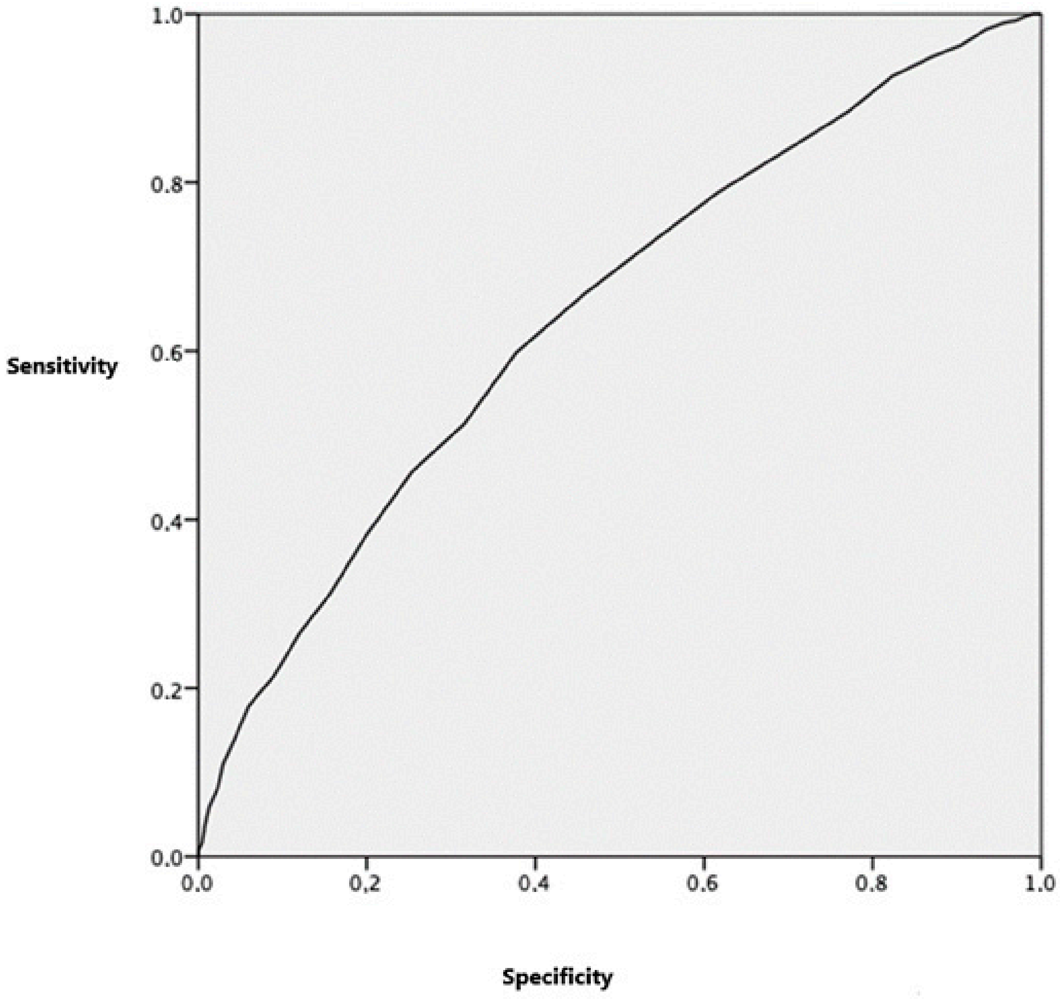


Figure S2. ROC MAGGIC CURVE
